# *Bordetella pertussis* Infection: From Immune Pathogenesis to Next-Generation Vaccines

**DOI:** 10.3390/vaccines14050384

**Published:** 2026-04-24

**Authors:** Vasiliki E. Georgakopoulou, Vassiliki C. Pitiriga

**Affiliations:** 1 Department of Pathophysiology, Laiko General Hospital, National and Kapodistrian University of Athens, 11527 Athens, Greece; 2 Department of Microbiology, Medical School of Athens, National and Kapodistrian University of Athens, 75 Mikras Asias Street, 11527 Athens, Greece

**Keywords:** *Bordetella pertussis*, pertussis pathogenesis, immune evasion, mucosal immunity, vaccine-induced immunity

## Abstract

Pertussis is a highly contagious respiratory infection caused by *Bordetella pertussis* and remains a persistent global health challenge despite widespread vaccination. This review aims to analyze the immune pathogenesis of *B. pertussis* infection and to identify key immunological limitations of current acellular pertussis vaccines that contribute to ongoing transmission. A narrative review of the literature was conducted, focusing on mechanisms of host–pathogen interaction, immune evasion, and vaccine-induced immunity. Evidence indicates that although acellular vaccines effectively reduce disease severity, they fail to prevent nasopharyngeal colonization and transmission, largely due to insufficient induction of mucosal immunity, T helper 1 (Th1) and T helper 17 (Th17) responses, and airway tissue-resident memory T cells. In contrast, natural infection induces broader immune responses, including secretory IgA production and robust cellular immunity, which are associated with improved bacterial clearance. Emerging next-generation vaccine strategies, including mucosal, outer membrane vesicle-based, and live-attenuated platforms, demonstrate enhanced ability to reduce bacterial colonization in preclinical and clinical models. In conclusion, effective control of pertussis transmission will require vaccine approaches that replicate infection-induced immunity at the respiratory mucosa, emphasizing the need for redesigned immunization strategies.

## 1. Introduction

Pertussis (whooping cough) is a highly contagious acute respiratory infection caused by the bacterium *Bordetella pertussis (B. pertussis*), an exclusively human pathogen. Despite widespread vaccination and significant reductions in incidence compared with the pre-vaccine era, pertussis remains a global health problem with cyclical outbreaks even in highly immunized populations [1,2]. In the absence of immunization, pertussis was historically a childhood disease with high morbidity and mortality; however, waning immunity following childhood vaccination has shifted a substantial burden to older age groups [3,4]. Immunity following acellular pertussis vaccination declines relatively rapidly, with observational data showing that protection diminishes within a few years after the last dose and may be limited beyond 7–10 years, contributing to increased susceptibility among adolescents and adults [3,5,6]. These older age groups now act as an important reservoir of infection, often transmitting *B. pertussis* to unvaccinated or partially vaccinated infants who are at the greatest risk of severe illness, hospitalization, and death [7].

Two main types of pertussis vaccines have been developed and widely implemented: whole-cell (wP) vaccines and acellular (aP) vaccines. Whole-cell vaccines, composed of inactivated *B. pertussis* organisms, were highly effective in reducing disease incidence but were associated with reactogenicity, leading to their replacement in many countries by acellular formulations. aP vaccines contain a limited number of purified antigens, including pertussis toxin (PT) (inactivated), filamentous hemagglutinin (FHA), pertactin, and fimbrial proteins, and are currently used in routine immunization schedules worldwide. While these vaccines have an improved safety profile and effectively protect against severe disease, accumulating evidence indicates that they do not induce durable immunity and fail to prevent nasopharyngeal colonization and transmission. This discrepancy between protection against clinical disease and inability to control infection represents a fundamental limitation of current vaccination strategies and provides the central rationale for the development of next-generation pertussis vaccines [3,5,6].

The pathogenesis of *B. pertussis* involves a coordinated array of adhesins and toxins that facilitate airway colonization and immune evasion. Key virulence factors include PT, FHA, pertactin and other adhesins, as well as adenylate cyclase toxin (ACT), which together modulate host defenses and promote bacterial persistence [2,8]. PT, in particular, disrupts G protein signaling in host cells through adenosine diphosphate (ADP)-ribosylation, subverting immune responses and contributing to disease manifestations [9]. Chemically inactivated PT is a central component of current acellular vaccines, which also include a limited set of additional antigens such as FHA, pertactin, and fimbriae [5,6]. Although these formulations induce strong antibody responses and reduce disease severity, they do not fully replicate the broad and durable immunity induced by natural infection or wP vaccines [3,10].

**Figure 1:**
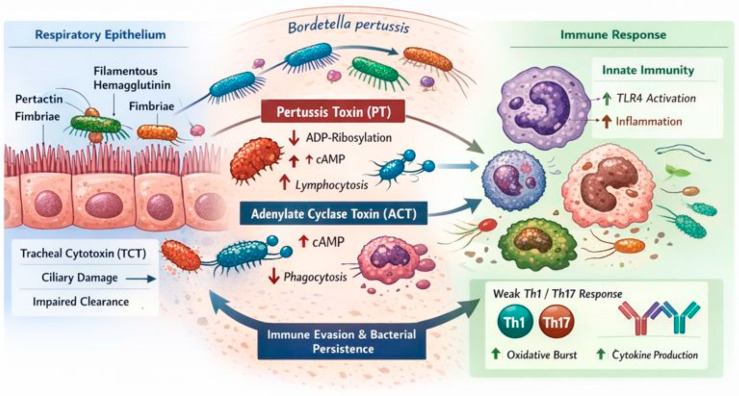
Pathogenesis and host immune modulation during *B. pertussis* infection. Following inhalation, *B. pertussis* adheres to the ciliated respiratory epithelium through surface adhesins, including filamentous hemagglutinin, pertactin, and fimbriae. After colonization, the bacterium produces multiple virulence factors that disrupt host defense mechanisms. Pertussis toxin (PT) induces ADP-ribosylation of inhibitory Gi proteins, leading to dysregulated intracellular cyclic adenosine monophosphate (cAMP) signaling, impaired leukocyte trafficking, and characteristic lymphocytosis. Adenylate cyclase toxin (ACT) enters phagocytic cells via complement receptor 3 (CD11b/CD18), causing pathological cAMP accumulation and suppression of phagocytosis and oxidative burst. Tracheal cytotoxin (TCT), a peptidoglycan fragment released during bacterial cell wall turnover, selectively damages ciliated epithelial cells, resulting in impaired mucociliary clearance. Although innate immune recognition through Toll-like receptor 4 (TLR4) activates inflammatory pathways, toxin-mediated immune modulation skews adaptive responses toward weak Th1 and Th17 immunity. Antibody responses limit disease severity but are insufficient to prevent bacterial persistence and transmission.

The key steps in the pathogenesis of *B. pertussis* infection and the associated host immune responses are summarized in Figure 1.

Clinically, pertussis progresses through three characteristic stages. The catarrhal stage presents with nonspecific symptoms including rhinorrhea, sneezing, and a mild persistent cough; this phase is highly infectious and may be indistinguishable from viral upper respiratory infections, particularly in infants who often lack the classic inspiratory “whoop” [2,7]. The paroxysmal stage follows, defined by recurrent, forceful coughing paroxysms that may be accompanied by vomiting, cyanosis, or hypoxia; in unvaccinated children, an inspiratory whoop is a recognized hallmark, and this stage carries the highest risk of complications including apnea and secondary bacterial pneumonia [7]. The convalescent stage involves a gradual decline in cough severity, although paroxysmal coughing can persist for weeks or progress to a chronic pattern.

Recent epidemiological data confirm a resurgence of pertussis in many regions, with increased incidence noted among adolescents and adults—populations with waning vaccine-derived immunity and under-recognized clinical disease [4,7,11]. These older individuals are increasingly identified as key contributors to transmission, particularly to young infants too young to complete primary immunization. As a result, many countries have expanded immunization strategies to include booster doses for older children, adolescents, and adults in an effort to reduce transmission and protect vulnerable infants [7,12].

Despite widespread vaccine use, pertussis continues to re-emerge because current acellular vaccines, although effective in reducing severe disease, do not reliably prevent upper-airway colonization, asymptomatic carriage, or transmission. This limitation indicates that the main challenge in pertussis vaccinology is no longer simply the prevention of clinical symptoms, but the induction of durable sterilizing immunity at the respiratory mucosa. Accordingly, the purpose of this review is not only to summarize the immune pathogenesis of *B. pertussis* infection, but to examine how the mechanistic failure of existing vaccines can inform the design philosophy of next-generation platforms. In this context, bacterial virulence factors, host immune dysregulation, and mucosal immune correlates are discussed as a framework for identifying the immune responses that future pertussis vaccines must reproduce in order to achieve durable, transmission-blocking protection.

This narrative review was conducted through a comprehensive literature search of the PubMed, Scopus, and Web of Science databases, focusing on studies addressing *B. pertussis* pathogenesis, host immune responses, and vaccine-induced immunity. Articles published in English without strict time restrictions were considered, with priority given to recent studies, systematic reviews, and key experimental investigations. Reference lists of relevant articles were also screened to identify additional sources. The selection aimed to provide a mechanistic and translational perspective linking immune pathogenesis with vaccine design.

Currently available pertussis vaccines fall into two principal categories: whole-cell pertussis (wP) vaccines and aP vaccines. wP vaccines consist of killed whole *B. pertussis* organisms and are still used in many national immunization programs, particularly in low- and middle-income countries, usually in combination with diphtheria and tetanus toxoids [1,2]. In contrast, aP vaccines contain purified pertussis antigens and are used predominantly in high-income countries in age-specific combinations such as DTaP for infants and young children and Tdap for adolescents, adults, and pregnant women [3,5,6]. Licensed acellular formulations differ in antigen composition, typically including inactivated PT, FHA, pertactin, and fimbrial proteins (FIM2/3) [5,6]. Three-component vaccines include PT, FHA, and pertactin, whereas five-component formulations additionally include fimbrial antigens [5,6]. Immunologically, although aP vaccines induce strong humoral responses and have an improved safety profile, they are associated with more rapid waning immunity and limited induction of Th1/Th17 and mucosal immune responses compared with natural infection or wP vaccines [3,5,6]. In contrast, wP vaccines induce broader and more durable immune responses, including stronger cellular immunity, albeit at the cost of higher reactogenicity [2,3]. These differences are central to the current resurgence of pertussis and highlight the need for next-generation vaccine strategies.

## 2. Bacterial Structure and Virulence Factors

The lipopolysaccharide (LPS) of *B. pertussis* represents a structurally atypical but immunologically active endotoxin that plays a central role in host–pathogen interactions and provides important insights into vaccine-induced immunity. Although *B. pertussis* LPS exhibits lower endotoxic activity than classical enterobacterial LPS, it is nevertheless recognized by the host innate immune system through Toll-like receptor 4 (TLR4), initiating downstream inflammatory signaling cascades [13,14]. The lipid A moiety of *B. pertussis* LPS is characterized by reduced phosphorylation and a distinct acylation pattern, features that attenuate excessive inflammation while preserving sufficient immunostimulatory capacity to activate innate immune pathways [15]. This finely balanced signaling appears critical for the induction of protective T-helper 1 (Th1)/T- helper 7 (Th17) responses during natural infection, which are notably underrepresented following immunization with current aP vaccines.

The limited ability of aP vaccines to reproduce infection-like innate immune activation represents a key mechanistic explanation for their suboptimal performance. In particular, alum-adjuvanted formulations predominantly promote Th2-skewed responses, in contrast to the TLR4-driven innate signaling required for effective Th1/Th17 polarization and mucosal immunity. Supporting this concept, synthetic lipidation of detoxified pertussis toxoid or incorporation of TLR agonists has been shown to restore innate immune activation and significantly enhance vaccine immunogenicity and protective efficacy, thereby bridging innate and adaptive immune responses [9,16,17].

Beyond LPS, *B. pertussis* deploys a repertoire of virulence factors that further illuminate the limitations of current vaccine strategies. PT and ACT are central to immune evasion, disrupting leukocyte trafficking and impairing phagocyte function through pathological elevation of intracellular cyclic adenosine monophosphate (AMP) [18,19]. While detoxified PT is included in aP vaccines and induces neutralizing antibodies, this antigen-specific approach does not fully replicate the complex immune activation induced during natural infection. In particular, the absence of robust cellular and mucosal immune responses—especially Th1/Th17 responses and tissue-resident memory T cells—may explain the inability of current vaccines to prevent nasopharyngeal colonization and transmission.

The structural and biological characteristics of *B. pertussis* further reinforce this concept. As a strictly human-adapted, Gram-negative coccobacillus with a reduced genome and specialized metabolic profile, the pathogen relies on tightly coordinated expression of toxins and adhesins, including pertactin and FHA, to establish infection within the respiratory mucosa [1,18,20]. However, current acellular vaccines include only a limited subset of these antigens, potentially restricting the breadth of immune recognition and contributing to incomplete protection against circulating strains.

Importantly, host–pathogen interactions mediated by factors such as ACT—through engagement with complement receptor 3 (CD11b/CD18)—highlight the critical role of early innate immune modulation in determining infection outcomes [19]. These mechanisms emphasize that effective vaccine-induced protection must extend beyond neutralizing antibodies to include coordinated innate activation, cellular immunity, and mucosal defense.

## 3. Adhesion and Colonization Mechanisms

Successful adhesion and colonization of the respiratory epithelium represent the critical first steps in *B. pertussis* infection and constitute a major barrier to the development of sterilizing vaccine-induced immunity. Multiple adhesins have been identified, although their precise hierarchy and regulation during human infection remain incompletely defined. The best-characterized include FHA, pertactin, fimbrial proteins (Fim2 and Fim3), and type IV pili [20,21,22]. Among these, FHA plays a dominant role in tracheal colonization by mediating high-affinity binding to ciliated epithelial cells via integrins and sulfated glycolipids [21]. Experimental studies have shown that immunization with FHA can reduce bacterial burden during early colonization, supporting its inclusion in acellular vaccine formulations [23]. However, despite the presence of FHA in current vaccines, protection against colonization remains incomplete, indicating that antigen-specific systemic immunity alone is insufficient to prevent mucosal infection.

Fimbriae contribute primarily to the initial stages of adherence and are tightly regulated by the BvgAS two-component virulence regulatory system, which coordinates the expression of colonization factors and toxins in response to environmental cues [20,24]. This coordinated regulation enables *B. pertussis* to rapidly adapt to the host environment and establish infection before effective immune clearance can occur. Following attachment, type IV pili and associated secretion systems facilitate bacterial persistence at the epithelial surface and may support limited internalization into host cells, although *B. pertussis* remains predominantly extracellular [25]. These features collectively promote sustained colonization of the respiratory mucosa, even in the presence of circulating antibodies.

A defining feature of *B. pertussis* infection is its strict localization to the human respiratory tract, where colonization of ciliated epithelial cells leads to the production of tracheal cytotoxin (TCT), a peptidoglycan fragment that selectively damages ciliated cells [26]. This results in impaired mucociliary clearance, facilitating bacterial persistence and promoting transmission through the characteristic paroxysmal cough. Importantly, these events occur at the mucosal surface, highlighting the limitations of systemic vaccine-induced immunity in controlling early infection dynamics.

Adhesion is mediated through multiple, partially redundant host–pathogen interactions. Early studies demonstrated selective binding of *B. pertussis* to ciliated respiratory epithelial cells, suggesting the involvement of specific host receptors [27]. Pertactin has been shown to interact with immunoglobulin-like domains, including IgD expressed on subsets of B cells, although no single receptor has been identified as essential for epithelial attachment [28]. This redundancy in adhesion mechanisms likely represents an evolutionary strategy to ensure robust colonization and may contribute to the limited effectiveness of vaccines targeting a restricted set of antigens. Furthermore, the precise adhesin–receptor interactions governing transmission between individuals remain incompletely understood, representing a critical gap in current knowledge.

## 4. Toxins Produced by *B. pertussis*: Implications for Immune Evasion and Vaccine Design

The toxins produced by *B. pertussis* are central to disease pathogenesis and, critically, provide mechanistic insight into why current vaccines fail to induce sterilizing immunity. Among these, PT and ACT are the most extensively characterized and biologically significant virulence factors [18,29]. Acting in a coordinated manner, these toxins disrupt both innate and adaptive immune responses, facilitate bacterial persistence within the respiratory tract, and promote transmission—features that are incompletely addressed by existing aP vaccines.

ACT, a bifunctional RTX family exotoxin, profoundly alters host cell signaling through dysregulation of intracellular cyclic AMP (cAMP). Following binding to complement receptor 3 (CD11b/CD18) on myeloid cells, ACT translocates its catalytic domain into the host cytosol, where it induces uncontrolled cAMP production, leading to protein kinase A activation and functional paralysis of mononuclear phagocytes [30,31]. This results in impaired phagocytosis, reduced oxidative burst, and suppressed cytokine production, allowing *B. pertussis* to evade early immune clearance [19]. These early innate immune disruptions are particularly important because they occur at the mucosal surface, where effective vaccine-induced protection would need to act rapidly to prevent colonization.

PT, a classical AB_5_ toxin, exerts systemic immunomodulatory effects through ADP-ribosylation of inhibitory Gi/Go G-proteins, leading to dysregulated G-protein–coupled receptor signaling and pathological accumulation of intracellular cAMP [32]. PT interferes with leukocyte trafficking, induces lymphocytosis, and suppresses effective immune responses, thereby contributing to disease severity and prolonged infection [29]. Although detoxified PT is a core component of aP vaccines and induces neutralizing antibodies, this antigen-specific response alone does not reproduce the broader immune activation required to control infection at the respiratory mucosa. This highlights a key limitation of current vaccine design, which relies heavily on systemic anti-toxin immunity while failing to induce robust mucosal and cellular responses [33,34].

In addition to PT and ACT, other virulence-associated factors—including FHA, fimbriae, and TCT—contribute to immune modulation, epithelial damage, and persistence within the respiratory tract [18,35]. These factors act synergistically to impair mucociliary clearance and establish a protected ecological niche that supports ongoing bacterial survival and transmission, even in the presence of circulating antibodies.

Genomic studies further reveal considerable genetic plasticity within circulating *B. pertussis* populations. Toxin-deficient or toxin-modified variants may emerge during infection, whereas vaccine formulations rely on stable, chemically inactivated toxin antigens [6]. This evolutionary divergence between circulating strains and vaccine components raises concerns regarding antigenic mismatch and highlights an additional limitation of current vaccines, which may not fully reflect the antigenic diversity encountered during natural infection [36,37].

Collectively, these observations indicate that toxin-mediated immune modulation is not only central to pathogenesis but also a key determinant of vaccine failure. Effective next-generation vaccines will therefore need to overcome these mechanisms by inducing rapid, localized, and multifunctional immune responses capable of counteracting toxin-driven immune suppression at the site of infection.

### 4.1. Pertussis Toxin (PT)

PT is widely regarded as the single most critical virulence determinant in the pathophysiology of pertussis and serves as a principal antigenic component of acellular pertussis vaccines [32,38]. PT is synthesized as a multimeric protein encoded by the ptx operon, which directs toxin production, post-translational modification, and secretion via the Ptl type IV secretion system [39]. Structurally, PT consists of an enzymatically active S1 subunit and a pentameric B oligomer responsible for host cell binding. Following internalization, the S1 subunit catalyzes ADP-ribosylation of Giα proteins, uncoupling inhibitory G-protein signaling and leading to persistent activation of adenylate cyclase with pathological increases in intracellular cAMP [32]. These effects disrupt immune cell migration, inhibit phagocytic responses, and promote lymphocytosis—one of the hallmark laboratory features of severe pertussis [29].

From a vaccine perspective, PT illustrates both the strengths and limitations of current antigen-specific strategies. While detoxified PT induces neutralizing antibodies that contribute significantly to protection against severe disease, these responses do not reliably prevent bacterial colonization or transmission. This discrepancy reflects the inability of PT-targeted immunity alone to reproduce the coordinated innate, cellular, and mucosal immune responses required for sterilizing protection at the respiratory epithelium [33,34]. Consequently, reliance on PT as a dominant vaccine antigen may contribute to the incomplete effectiveness of current acellular vaccines.

Genetic instability of the ptx operon has been described in laboratory-passaged *B. pertussis* strains, particularly in the reference strain Tohama I, where spontaneous mutations or loss of operon function may occur during repeated subculturing [40]. Such instability complicates in vitro toxin production and has historically necessitated careful strain selection for vaccine antigen manufacture. Importantly, although PT-deficient mutants may emerge experimentally or transiently during infection, circulating clinical isolates generally retain functional toxin expression, highlighting the essential role of PT in bacterial fitness and transmission [6,41].

Beyond its direct pathogenic effects, PT may also contribute indirectly to bacterial survival by modulating host–microbe interactions and microbial competition within the respiratory tract, although these ancillary roles remain incompletely understood [17]. Overall, PT exemplifies the need for vaccine strategies that extend beyond neutralizing toxin activity to include broader immune activation capable of controlling early infection and transmission dynamics.

### 4.2. Adenylate Cyclase Toxin (ACT)

ACT, encoded by the cyaA gene, is another major virulence factor expressed by *B. pertussis*, as well as *B. parapertussis* and *B. bronchiseptica* [30]. ACT is a large (~1706 amino acid) multifunctional protein comprising an N-terminal calmodulin-dependent adenylate cyclase domain and a C-terminal Repeats-in-Toxin (RTX) hemolysin domain responsible for membrane interaction and translocation [31,42].

The N-terminal catalytic domain is activated upon binding host calmodulin, enabling rapid and unregulated cAMP synthesis in intoxicated cells. The large size and modular architecture of ACT facilitate efficient translocation across host cell membranes, an essential determinant of its biological potency [43]. Post-translational palmitoylation by the CyaC acyltransferase is required for full toxin activity and effective host cell targeting [44].

Although ACT is not essential for lethality in murine respiratory infection models, it plays a critical role during early infection by suppressing innate immune responses and enhancing bacterial colonization efficiency [19,45]. By impairing phagocyte function at the site of infection, ACT contributes to early immune evasion, a phase during which vaccine-induced protection must act rapidly to prevent establishment of infection.

Importantly, current acellular pertussis vaccines do not directly target ACT-mediated immune suppression. This represents a significant gap in vaccine-induced immunity, as early innate immune dysregulation may permit bacterial colonization even in vaccinated individuals. Distinct structural variants of ACT have been described across Bordetella species, with virulent *B. pertussis* strains typically producing a full-length, highly active toxin, whereas other species may generate truncated or alternative forms with differing biological activity [42].

### 4.3. Tracheal Cytotoxin (TCT)

TCT is a key virulence factor of *B. pertussis* that contributes directly to epithelial damage and disruption of mucociliary clearance, a defining pathological feature of pertussis. Unlike classical protein exotoxins, TCT is a low-molecular-weight peptidoglycan fragment released during bacterial cell wall turnover and is not actively secreted through specialized secretion systems [35,46]. Chemically, TCT corresponds to a disaccharide–tetrapeptide monomer of peptidoglycan (N-acetylglucosamine–N-acetylmuramic acid–tetrapeptide) with a molecular mass of approximately 921 Da, rather than a lipid-based structure [47].

Production and accumulation of TCT are influenced by environmental conditions, including nutrient availability. Experimental data indicate that iron-limited conditions—resembling the host respiratory tract—favor increased release of peptidoglycan fragments, including TCT, whereas iron-replete environments suppress their accumulation [48]. This regulatory pattern likely enhances pathogenic effects specifically within the human airway, where bacterial survival depends on efficient disruption of epithelial defense mechanisms.

TCT selectively targets ciliated epithelial cells of the trachea and bronchi, inducing nitric oxide synthase expression and excessive nitric oxide production, which leads to oxidative stress, ciliary dysfunction, and eventual ciliated cell extrusion [49]. These effects result in impaired mucociliary clearance and create a permissive niche for bacterial persistence, thereby facilitating prolonged infection and efficient transmission. Importantly, these events occur at the mucosal surface, highlighting a critical limitation of current acellular vaccines, which predominantly induce systemic immunity but do not provide sufficient protection at the site of initial infection.

Unlike PT, TCT does not appear to significantly modulate systemic anti-PT antibody responses [29]. However, immune recognition of TCT at the mucosal surface plays an important role in limiting bacterial attachment during early exposure. High levels of TCT-specific IgA and IgM have been detected in nasal secretions following exposure, suggesting that mucosal antibodies act as a first-line barrier preventing epithelial damage and reducing effective colonization [50]. Subsequent systemic IgG and IgA responses contribute to longer-term immune control, with no evidence of antigenic variation in TCT that would compromise antibody binding [51]. These observations indicate that TCT is a stable and conserved target, further supporting its potential relevance in vaccine design.

Importantly, primary exposure to *B. pertussis* may remain clinically silent, particularly in partially immune individuals, yet such asymptomatic or minimally symptomatic carriers can serve as major reservoirs for transmission within susceptible populations [52]. TCT-mediated epithelial injury is thought to facilitate bacterial shedding and environmental dissemination, reinforcing its role not only in pathogenesis but also in epidemiological spread [49,53]. Experimental models have demonstrated that neutralization of TCT—particularly at the mucosal surface—reduces epithelial damage and limits bacterial transmission [29,50].

### 4.4. Human Carriage and Controlled Human Infection Models

In response to the growing recognition that transmission is frequently driven by minimally symptomatic or asymptomatic carriage, controlled human infection models (CHIMs) have been developed to safely induce nasopharyngeal colonization in healthy adults under intensive monitoring. These models provide a unique opportunity to study early host–pathogen interactions and define immune correlates of protection at the site of infection.

In the PERISCOPE program, intranasal inoculation with a well-characterized clinical isolate (B1917) reproducibly established colonization without classical pertussis symptoms. Colonization and shedding were quantified through serial sampling, including pernasal and nasopharyngeal swabs, nasal washes, and throat swabs, combined with culture and quantitative polymerase chain reaction (PCR). Eradication therapy (e.g., azithromycin) was administered at predefined timepoints to terminate carriage, ensuring participant safety while enabling detailed analysis of colonization dynamics [54,55].

These platforms are particularly valuable for next-generation vaccine development because they directly measure the key outcome that current acellular vaccines fail to control: acquisition of colonization and bacterial burden in the upper airway. The most advanced application to date is the BPZE1 challenge program (CHAMPION-1), in which participants received a single intranasal dose of live-attenuated BPZE1 or placebo and were subsequently challenged with virulent *B. pertussis* under controlled conditions. Using repeated quantitative culture and qPCR measurements, BPZE1 reduced both the proportion of volunteers with detectable colonization and the overall bacterial load compared with placebo [37,45]. These findings provide direct human evidence that mucosal, infection-like immune responses can reduce carriage and may represent a critical requirement for next-generation vaccine efficacy.

Methodologically, CHIMs also provide an experimental framework to refine sampling strategies and microbiological quantification. In addition to standard culture and DNA-based qPCR, viability-linked approaches such as propidium monoazide pre-treatment coupled with qPCR can distinguish viable from non-viable organisms and may better approximate infectious shedding [56].

All controlled human infection studies are conducted under strict ethical oversight, including informed consent, predefined eradication protocols, and intensive clinical monitoring to ensure participant safety.

## 5. Immune Response to *B. pertussis*

Protective immunity against *B. pertussis* is complex and multifactorial, requiring coordinated activation of innate, humoral, and cellular immune responses. Naturally acquired infection induces broader and more durable immunity than vaccination—particularly when compared with aP vaccines—although neither form of immunity is lifelong [33,34]. Effective control of bacterial replication and prevention of persistent colonization depend on the integration of antibody-mediated protection with robust T-cell–dependent immune mechanisms [36]. Importantly, the inability of current vaccines to reproduce this coordinated immune response represents a central limitation underlying their failure to prevent infection and transmission.

Antibodies play a critical role in limiting disease severity by neutralizing PT, inhibiting bacterial adhesion, and promoting opsonophagocytic clearance. However, humoral immunity alone is insufficient to prevent infection or transmission. Experimental and clinical data demonstrate that effective antibody responses require appropriate innate immune signaling—particularly through pattern recognition receptors such as TLR4—to support T-helper cell differentiation and durable immunological memory [17,37]. In the absence of such signals, antibody production may occur but lacks functional efficacy and long-term protective capacity, a limitation characteristic of current acellular vaccine formulations.

During *B. pertussis* infection, the innate immune system responds rapidly to bacterial components including LPS, toxins, and adhesins. These factors activate epithelial cells, macrophages, dendritic cells, and neutrophils, initiating cytokine and chemokine cascades that shape downstream adaptive responses [19]. However, *B. pertussis* has evolved mechanisms to subvert these pathways. PT and ACT impair phagocytic killing, disrupt antigen presentation, and skew T-cell polarization away from protective Th1/Th17 responses, thereby facilitating sustained bacterial survival at the respiratory mucosa [17,57,58,59]. These early disruptions are particularly relevant for vaccine design, as they occur at the site and timing where immune responses must act to prevent colonization.

Cell-mediated immunity, particularly Th1- and Th17-type responses, is essential for bacterial clearance. Interferon-γ–producing Th1 cells promote macrophage activation, while Th17 cells enhance neutrophil recruitment and strengthen mucosal defense [60]. In contrast, acellular pertussis vaccines predominantly induce Th2-skewed responses, with relatively weak induction of Th1/Th17 immunity. This imbalance contributes directly to the failure to achieve sterilizing immunity and explains the persistence of asymptomatic carriage and ongoing transmission in vaccinated populations [34,60].

Taken together, these findings highlight a fundamental mismatch between the immune responses induced by natural infection and those elicited by current vaccines. While natural infection generates integrated innate activation, mucosal immunity, and Th1/Th17 polarization, acellular vaccines primarily induce systemic antibody responses with limited mucosal and cellular components. This discrepancy provides a mechanistic basis for vaccine failure and underscores the need for next-generation vaccine strategies that can induce durable, infection-like immune responses at the respiratory epithelium.

## 6. Cellular Interactions During Infection

Direct interactions between *B. pertussis* and the respiratory epithelium are central to colonization and disease initiation. The bacterium preferentially binds to ciliated epithelial cells lining the upper and lower airways, where it establishes a localized extracellular niche [61]. These early interactions define the initial stages of infection and represent a critical window during which effective immune responses must act to prevent bacterial persistence.

Adhesion to epithelial surfaces is mediated by a repertoire of surface-expressed adhesins, including FHA, fimbriae (Fim2 and Fim3), and pertactin [21]. These adhesins facilitate stable attachment, resist mechanical clearance by mucociliary activity, and promote bacterial persistence. FHA plays a dominant role through interactions with integrins and sulfated glycoconjugates on epithelial and immune cells, while fimbriae contribute to early-stage attachment and colonization [62]. However, despite the inclusion of some of these antigens in acellular vaccines, effective prevention of colonization is not achieved, further emphasizing that antigen-specific systemic immunity alone is insufficient to block mucosal infection.

Beyond epithelial cells, *B. pertussis* interacts dynamically with immune cells such as macrophages and dendritic cells. Although the bacterium is primarily extracellular, limited internalization into host cells has been observed and may contribute to immune modulation rather than intracellular replication [53]. These interactions influence antigen presentation, cytokine secretion, and the quality of the adaptive immune response, reinforcing the importance of early host–pathogen interactions in shaping disease outcome.

Despite extensive research, the precise molecular determinants governing epithelial tropism, host specificity, and efficient transmission—particularly in adult-to-adult and adult-to-infant spread—remain incompletely understood [18,61]. This knowledge gap has important implications for vaccine development, as incomplete understanding of early colonization mechanisms may limit the ability to design vaccines capable of fully preventing infection and transmission.

## 7. Inflammatory Response in Pertussis

The inflammatory response to *B. pertussis* infection is characterized by a distinctive imbalance between pronounced local airway inflammation and relatively modest systemic manifestations. Lung inflammation is driven by activation and subsequent dysregulation of neutrophils, alveolar macrophages, and epithelial cells, which collectively orchestrate both innate and adaptive immune responses [19,43]. Notably, this inflammatory response is not effectively resolved, reflecting the ability of *B. pertussis* to simultaneously trigger and subvert host immunity.

A hallmark of pertussis pathology is extensive leukocyte activation followed by immune cell dysfunction and death. Neutrophils and macrophages that internalize *B. pertussis* are exposed to ACT-mediated cAMP overload and PT–induced signaling disruption, resulting in impaired chemotaxis, defective phagolysosomal function, and eventual cell lysis [29,37]. The accumulation and subsequent destruction of these immune cells contribute to local tissue damage, airway inflammation, and impaired gas exchange, which may underlie the mild hypoxia observed during infection despite preserved ventilatory drive [63].

Emerging evidence further implicates dysregulation of intracellular calcium (Ca^2+^) homeostasis as a key mechanism underlying immune cell dysfunction. Disruption of Ca^2+^ balance impairs actin cytoskeleton dynamics in phagocytes, limiting motility, chemotaxis, and effective immune surveillance [64]. In neutrophils with compromised phagocytic capacity, this imbalance exacerbates inflammatory injury while paradoxically reducing bacterial clearance. These findings highlight a central feature of pertussis pathophysiology: inflammation that is both excessive and functionally ineffective.

Importantly, this dysregulated inflammatory response occurs predominantly at the respiratory mucosa, where early immune control is required to prevent colonization. The inability of current acellular vaccines to induce effective mucosal immunity or rapidly deploy functional innate responses at this site may contribute to their failure to prevent infection and transmission.

## 8. Immune Pathogenesis of *B. pertussis*

The immune pathogenesis of *B. pertussis* infection is defined by the paradoxical coexistence of intense immune activation and profound immune dysfunction. Rather than evading immune recognition entirely, *B. pertussis* actively manipulates innate and adaptive immune responses to favor prolonged survival in the respiratory tract, persistent inflammation, and efficient transmission [18,63]. Disease severity and the failure to achieve sterilizing immunity are therefore driven primarily by immune dysregulation rather than uncontrolled bacterial replication.

Initial host–pathogen interactions occur at the respiratory mucosa, where epithelial cells and resident macrophages recognize bacterial components such as LPS, peptidoglycan fragments, and adhesins via pattern-recognition receptors, particularly TLR4 [34]. This recognition initiates early inflammatory signaling and cytokine production; however, *B. pertussis* rapidly subverts these responses through the coordinated action of PT, ACT, and TCT [17].

PT plays a central role in systemic immune dysregulation. By ADP-ribosylating inhibitory Gi proteins, PT disrupts G-protein–coupled receptor signaling, leading to aberrant intracellular cAMP accumulation in immune cells [65]. This impairs chemokine receptor function, inhibits leukocyte extravasation, and results in the characteristic lymphocytosis observed in severe pertussis [66]. Consequently, immune cells are abundant in circulation but functionally excluded from sites of infection, delaying effective bacterial clearance.

ACT further amplifies immune dysfunction at the local level by delivering a calmodulin-dependent adenylate cyclase into phagocytes. The resulting supraphysiologic cAMP concentrations suppress phagocytosis, oxidative burst, antigen presentation, and proinflammatory cytokine production in neutrophils, macrophages, and dendritic cells [19]. These effects compromise both innate bacterial killing and the priming of adaptive immune responses during the critical early stages of infection.

Simultaneously, TCT induces targeted destruction of ciliated epithelial cells through nitric oxide-mediated cytotoxicity, impairing mucociliary clearance and perpetuating bacterial persistence [67]. This epithelial injury sustains chronic airway inflammation while facilitating ongoing bacterial shedding and transmission.

Adaptive immunity is similarly shaped by toxin-mediated immune modulation. Effective bacterial clearance requires coordinated Th1 and Th17 responses, which promote macrophage activation and neutrophil recruitment at the mucosal surface [59]. However, PT and ACT disrupt T-cell polarization, limiting the development of durable Th1/Th17 immunity and favoring suboptimal responses that permit persistent colonization and asymptomatic carriage [68]. This phenomenon is particularly evident following acellular pertussis vaccination, which induces strong humoral responses but relatively weak cellular immunity, thereby failing to prevent colonization and transmission despite protecting against severe disease [33].

Humoral immunity remains essential for limiting pathology by neutralizing toxins and reducing disease severity. However, antibodies alone are insufficient for sterilizing immunity. Effective antibody responses depend on intact innate signaling pathways—particularly TLR4-mediated activation—which are both dampened during infection and inadequately stimulated by acellular vaccines [34,69]. This incomplete immune activation provides a mechanistic explanation for the limited durability and functional efficacy of vaccine-induced protection.

Collectively, these findings demonstrate that *B. pertussis* pathogenesis is driven by coordinated immune manipulation at both the systemic and mucosal levels. This dual disruption highlights a fundamental limitation of current vaccine strategies and underscores the need for next-generation vaccines capable of inducing rapid, localized, and functionally integrated immune responses that overcome toxin-mediated immune suppression.

## 9. Immune Pathogenesis as a Framework for Next-Generation Vaccine Design

The key immunopathogenesis insight driving “next-generation” pertussis vaccine design is that current acellular pertussis (aP) vaccines protect well against symptomatic disease but do not reliably prevent nasopharyngeal colonization and onward transmission, largely because they fail to elicit durable mucosal immunity and robust Th1/Th17 cellular responses in the respiratory tract [33,70,71]. Natural infection (and, to a greater extent, older whole-cell vaccines) induces a broader immune program that includes Th1/Th17 polarization, airway tissue-resident memory T cells (T_RM), and secretory IgA, which are repeatedly implicated as requirements for sterilizing immunity at the site of entry [33,50,70,71].

### 9.1. Redesign Target: Block Colonization and Transmission (Mucosal Immunity, T_RM, sIgA)

*B. pertussis* largely remains localized to the airways, and its toxins (PT, ACT) and inflammatory injury (including TCT-mediated epithelial damage) create a setting where systemic antibodies can limit severity but do not necessarily clear carriage [33,71]. This shifts the primary vaccine goal from “prevent disease” to “prevent infection/colonization in the nose and upper airway.” Reviews synthesizing animal and human data emphasize that mucosal Th17 responses, interleukin-17 (IL-17)-driven recruitment, and sIgA, alongside respiratory T_RM, are central correlates for durable anti-colonization immunity—precisely the compartments that aP vaccines weakly induce [50,70].

Consistent with this mechanistic goal, intranasal vaccination strategies have shown improved control of upper-airway colonization in preclinical models. Intranasal delivery of acellular antigens can induce *B. pertussis*–specific mucosal IgA and IL-17A and reduce bacterial burden in both upper and lower respiratory tracts in mice, demonstrating that “route” can partially correct the immunologic deficit of standard intramuscular aP vaccination [72].

In addition to antigen composition, the route of vaccine delivery plays a critical role in shaping the quality and localization of the immune response. Intramuscular administration, as used in current acellular pertussis vaccines, primarily induces systemic humoral immunity characterized by circulating IgG antibodies, which are effective in reducing disease severity but have limited impact on bacterial colonization at the respiratory mucosa [33,70,71]. In contrast, intranasal vaccination directly targets the site of infection and has been shown to induce mucosal immune responses, including secretory IgA production, Th17 polarization, and the generation of airway tissue-resident memory T cells [50,72]. These immune components are increasingly recognized as essential for preventing nasopharyngeal colonization and interrupting transmission [33,70,71]. Comparative studies in animal models demonstrate that intranasal delivery can significantly reduce bacterial burden in both the upper and lower respiratory tract, whereas intramuscular vaccination fails to achieve sterilizing immunity despite inducing robust systemic antibody responses [2,72]. These findings highlight that the route of immunization is not merely a technical parameter, but a key determinant of vaccine effectiveness in the context of pertussis transmission dynamics.

### 9.2. Redesign Target: Restore Th1/Th17-Skewing Through Innate-Programming Adjuvants

Acellular vaccines are classically alum-adjuvanted and tend to skew toward Th2-biased responses, whereas clearance of *B. pertussis* and prevention of carriage are more strongly associated with Th1/Th17 immunity [33,70,71]. This has motivated redesign around pattern-recognition receptor (PRR) agonists that better mimic infection-like innate signals.

A practical proof-of-concept is that adding a TLR7 agonist to an aP formulation significantly enhances Th1/Th17 responses and protection in a mouse model, directly aligning vaccine outputs with the immune mechanisms required for bacterial control at mucosal surfaces [2]. TLR4 agonist biology is also central to modern vaccinology: monophosphoryl lipid A (MPL) is a TRIF-biased TLR4 agonist that supports potent adjuvant effects with improved tolerability relative to native lipid A, providing a mechanistic foundation for TLR4-oriented adjuvant strategies in pertussis vaccine redesign [73].

### 9.3. Redesign Target: Broaden Antigenic Breadth and Present Antigens in “Pathogen-Like” Formats (OMV, Biofilm/Clinical Isolate Relevance)

Another immunopathogenesis-informed limitation of current aP vaccines is their narrow antigen set and potential mismatch with evolving circulating strains, alongside suboptimal induction of mucosal cellular immunity [33,70,71]. To address this, outer membrane vesicle (OMV)-based vaccines aim to present multiple antigens in their native membrane context (including innate-stimulatory components), thereby improving the breadth and functional quality of immunity.

OMV-based pertussis vaccine candidates have demonstrated long-lasting protection and cross-protection across strain genotypes in preclinical work [74]. Critically, intranasal OMV vaccination has been shown to induce mucosal IgA and Th17-mediated responses and—most importantly—prevent colonization in both lungs and nasal cavity, which maps directly onto the “stop transmission” design target derived from immune pathogenesis [75].

### 9.4. Redesign Target: Consider Live-Attenuated Nasal Vaccines to Recapitulate Infection-Like Immunity Safely

Because natural infection induces the most complete mucosal immune program (including airway T_RM), live-attenuated nasal vaccines are a rational extension of immune-pathogenesis principles—provided safety and genetic stability are addressed [50,70]. BPZE1 is a leading example, designed to induce both mucosal and systemic immunity. Human clinical data have now advanced substantially: BPZE1 has demonstrated immunogenicity and acceptable safety in adults in clinical studies [76], and a controlled human infection model phase 2b trial reported that a single nasal administration could prevent or substantially reduce colonization by virulent *B. pertussis*—a direct test of the transmission-blocking goal [77].

### 9.5. Structured Comparison of Next-Generation Pertussis Vaccine Platforms

To operationalize the immune-pathogenesis blueprint, Table 1 contrasts major vaccine platforms using a consistent set of immune correlates that are repeatedly linked to sterilizing immunity in the upper airway (mucosal IgA, Th1/Th17 polarization, and airway tissue-resident memory T cells), together with the most policy-relevant outcome: prevention of nasopharyngeal colonization and onward transmission.

Currently marketed *B. pertussis* vaccines consist of wP and aP formulations [2,38]. In the research pipeline, next-generation candidates include intranasal subunit/aP-based vaccines [2,73], outer membrane vesicle (OMV)-based vaccines [74,75], and the live-attenuated nasal vaccine BPZE1 [76,78]. Among these investigational approaches, BPZE1 has progressed to human clinical trials, including phase 2b studies, whereas OMV-based and most intranasal subunit candidates remain at the preclinical stage [74,75,76,77].

### 9.6. Epitope-Based Vaccine Design: Toward Precision Immunization

Recent advances in immunoinformatics and structural vaccinology have highlighted the potential of epitope-based vaccine design as a complementary strategy for next-generation pertussis vaccines [78,79,80,81]. In contrast to conventional acellular vaccines, which rely on a limited number of full-length antigens, epitope-based approaches aim to identify and incorporate discrete immunodominant and functionally protective regions of key virulence factors, including PT, FHA, pertactin, and ACT [78,79,80,81].

Protective epitopes within PT are primarily associated with neutralizing antibody responses targeting domains involved in receptor binding and enzymatic activity, which are critical for toxin function [82,83]. Similarly, mapped epitopes within FHA and pertactin include regions implicated in bacterial adhesion and antibody-mediated inhibition of colonization, supporting their continued relevance as precision antigen targets [82,83,84,85]. In parallel, the identification of T-cell epitopes capable of promoting Th1- and Th17-skewed responses is of particular importance, given the central role of these immune pathways in bacterial clearance and mucosal defense [70,71,86].

A key advantage of epitope-based vaccine design is the ability to prioritize conserved antigenic regions across circulating *B. pertussis* strains, thereby potentially reducing the impact of antigenic variation and improving cross-protective immunity [34,36,78]. Moreover, rational epitope selection may enable simultaneous targeting of multiple stages of infection, including bacterial adhesion, immune evasion, and toxin-mediated pathology, thereby addressing several mechanistic limitations of current acellular vaccines [78,79,84].

Importantly, epitope-based strategies must be integrated with appropriate delivery systems and adjuvants to achieve effective immune activation. In particular, coupling selected epitopes with mucosal delivery platforms or pattern-recognition receptor agonists may enhance Th1/Th17 polarization and improve immune targeting at the respiratory mucosa [60,70,71,72,73,74]. Whether such approaches can reliably induce airway tissue-resident memory T cells and robust secretory IgA responses remains to be fully established; however, they provide a rational and flexible framework for precision vaccine design aimed at overcoming the key immunological limitations of existing pertussis vaccines [60,70,71].

An important consideration for next-generation pertussis vaccines is the durability of mucosal and tissue-resident immune responses, as well as their long-term impact on transmission dynamics and herd immunity at the population level.

Table 2 summarizes the currently available pertussis vaccines and their principal characteristics.

## 10. Resistance to Antibiotics in *B. pertussis*

Macrolides have long represented the cornerstone of both treatment and post-exposure prophylaxis for *B. pertussis* infection. Erythromycin, azithromycin, and clarithromycin are recommended as first-line agents owing to their activity against *B. pertussis*, favorable penetration into respiratory secretions, and demonstrated efficacy in shortening infectiousness and reducing secondary transmission [87,88]. For decades, antimicrobial resistance in *B. pertussis* was considered negligible; however, over the past fifteen years, the emergence and expansion of macrolide-resistant *B. pertussis* (MRBP) has fundamentally altered this paradigm.

### 10.1. Molecular Mechanisms of Macrolide Resistance

Macrolide resistance in *B. pertussis* is now well characterized at the molecular level and is primarily mediated by a point mutation in domain V of the 23S rRNA gene, most commonly the A2047G substitution (Escherichia coli numbering) [89]. This mutation induces a conformational alteration of the ribosomal macrolide-binding site, effectively preventing antibiotic binding and resulting in high-level resistance. Phenotypically, resistant isolates typically exhibit minimum inhibitory concentrations (MICs) exceeding 256 μg/mL for erythromycin, azithromycin, and clarithromycin, levels far above achievable serum or tissue concentrations [90,91].

Importantly, *B. pertussis* possesses three copies of the 23S rRNA gene, and high-level resistance generally requires mutation of all operons. This biological constraint likely explains the historical rarity of resistance and underscores why macrolide resistance emerged relatively late despite widespread antibiotic use. Once resistant clones with full operon mutations became established, however, rapid clonal expansion has been observed [41]. Notably, standardized interpretive criteria for antimicrobial susceptibility testing of *B. pertussis* have not been established by either CLSI or EUCAST, and confirmation of resistance relies on molecular detection of resistance mutations or demonstration of exceptionally high MICs in vitro [92].

### 10.2. Global Epidemiology of Macrolide-Resistant B. pertussis

China has emerged as the global epicenter of MRBP. Following the first reports of macrolide-resistant isolates in 2011, surveillance studies have demonstrated a dramatic and sustained increase in prevalence, with resistance rates exceeding 80–90% in multiple regions and approaching fixation in some northern provinces [89,92]. This extraordinary prevalence is widely attributed to intense selective pressure from macrolide overuse, combined with clonal dissemination of resistant lineages. In such settings, macrolide therapy is likely to be ineffective for both treatment and prophylaxis, substantially undermining outbreak control efforts.

Outside China, MRBP has been reported less frequently but across an expanding geographic range. In Japan, resistant isolates have been increasingly detected, including a fatal infant case confirmed to be caused by macrolide-resistant *B. pertussis*, underscoring the potential clinical consequences of resistance in high-risk populations [93]. In Europe, resistance remains rare but is no longer confined to isolated detections; Finland reported a resistant isolate in national surveillance in 2024, and France documented localized clusters during the same period [94]. These findings indicate that MRBP is emerging within Europe, even if prevalence remains low at present.

In the Americas, reports of MRBP have been historically sporadic; however, recent regional alerts have documented resistant isolates in several countries, including Brazil, Mexico, Peru, and the United States, suggesting wider circulation than previously appreciated [95]. Similarly, hospital-based surveillance in Hong Kong during 2023–2024 revealed a predominance of resistant strains among circulating *B. pertussis* isolates, raising concern that localized dominance of MRBP may develop outside mainland China under favorable conditions [96].

## 11. Clinical and Public Health Implications

The emergence of MRBP poses significant clinical and public health challenges. Although vaccination remains the primary tool for pertussis prevention, breakthrough infections occur due to waning immunity and incomplete protection against colonization. In this context, effective antimicrobial therapy is essential for reducing disease severity, preventing transmission, and protecting vulnerable contacts, particularly infants. Macrolide resistance eliminates the effectiveness of all first-line agents, as resistant strains demonstrate cross-resistance to erythromycin, azithromycin, and clarithromycin [89].

Trimethoprim–sulfamethoxazole (TMP-SMX) remains the only validated alternative therapy and is recommended for patients aged ≥2 months when macrolide resistance is suspected or confirmed [87]. However, TMP-SMX is contraindicated in neonates and during pregnancy, precisely the populations at highest risk for severe pertussis outcomes. Although fluoroquinolones and tetracyclines exhibit in vitro activity against *B. pertussis*, their use is limited by safety concerns and the absence of robust clinical efficacy data, particularly in children [91].

Beyond individual patient management, MRBP threatens the effectiveness of chemoprophylaxis strategies in households, neonatal units, and healthcare settings. Failure to eradicate carriage in index cases increases the risk of sustained transmission, potentially amplifying outbreaks even in highly vaccinated populations.

Given that MRBP is largely driven by a ribosomal target-site mutation, a complementary strategy is systematic in vitro screening of (i) newer macrolide derivatives (e.g., ketolides) with improved ribosomal interactions and (ii) adjuvant compounds that increase intracellular drug exposure (e.g., efflux pump inhibitors or membrane-permeabilizing potentiators) in combination with macrolides. Standard checkerboard assays and time–kill kinetics against contemporary MRBP and macrolide-susceptible isolates, alongside 23S rRNA genotyping, would enable quantification of synergy [fractional inhibitory concentration, (FIC) indices] and selection of candidates for in vivo validation.

In regions where MRBP prevalence is high or during outbreaks with confirmed A2047G strains, management should incorporate (a) rapid molecular resistance detection when available (e.g., 23S rRNA domain V genotyping), (b) age- and pregnancy-specific selection of the best-supported alternative agents, and (c) explicit acknowledgement of evidence gaps (especially neonates and pregnancy). Table 3 summarizes pragmatic options aligned with existing guidance and the limited MRBP-specific clinical evidence base [57,66,96,97,98,99,100,101].

Because clinical benefit from antibiotics in pertussis is greatest when given early and because antimicrobial therapy is also used to reduce infectiousness, the emergence of MRBP creates a disproportionately high-risk scenario for groups in whom (i) bacterial burden is highest (young infants), (ii) complications are more frequent (pregnancy-related exposure of neonates; immunocompromised hosts), and (iii) validated non-macrolide options are restricted by safety.

## 12. Implications for Surveillance, Stewardship, and Prevention

The emergence and expansion of MRBP highlight the urgent need for enhanced pertussis surveillance incorporating molecular resistance detection and genomic epidemiology. Routine sequencing of circulating isolates would enable early identification of resistant clones and facilitate tracking of their spread at national and international levels [41,94]. Treatment guidelines may require revision in regions where resistance becomes prevalent, balancing the use of alternative agents against safety considerations.

From an antimicrobial stewardship perspective, the Chinese experience serves as a cautionary example of how high antibiotic pressure can rapidly select for and entrench resistance in *B. pertussis*. Judicious macrolide use, combined with robust vaccination programs, is likely to be essential in preventing similar scenarios elsewhere [92]. Given increasing global mobility, international coordination in surveillance and reporting will be critical to limit dissemination of resistant strains.

## 13. Emerging Issues and Research Directions

A growing body of experimental and clinical evidence indicates that exposure of *B. pertussis* to host immune serum induces profound phenotypic and functional changes, underscoring the dynamic nature of host–pathogen interactions and revealing underexplored mechanisms of immune-mediated bacterial modulation. Serum-exposed *B. pertussis* organisms display altered expression of multiple surface-associated factors, including reduced hemadsorptive fimbrial activity, diminished expression of phase I virulence components, and impaired attachment to ciliated respiratory epithelial cells [95,96]. These observations suggest that humoral immune pressure not only neutralizes bacterial toxins but can directly interfere with colonization capacity, a concept with important implications for vaccine-induced protection.

Early immunological studies demonstrated that serum exposure leads to the release or downregulation of surface components involved in adhesion and immune evasion, highlighting a potential antibody-driven “phenotypic silencing” of virulence traits [95]. Among the immune-reactive components identified, the so-called leukocytosis-promoting factor—now recognized as PT—was shown to induce lymphocyte activation and serum agglutinins capable of protecting mice against intranasal challenge [102]. Importantly, reciprocal protection models established that serum agglutinins and PT-mediated immune effects represent independent mechanisms of protection, emphasizing the multiplicity of immune pathways involved in host defense [63].

Species-specific differences in lymphocyte responsiveness to *B. pertussis* antigens further highlight the complexity of immune recognition. While human, canine, and primate lymphocytes exhibit proliferative or agglutinating responses following exposure to PT or immune serum, bovine lymphocytes do not, suggesting host-restricted immune pathways shaped by co-evolution with an exclusively human pathogen [65]. Notably, optimal lymphocyte responses in experimental systems were achieved in the presence of non-specific mitogens such as concanavalin A, pokeweed mitogen, or Bacillus Calmette–Guérin (BCG), rather than *Bordetella*-specific antigens alone, underscoring the importance of innate immune co-stimulation in shaping effective adaptive responses [63]. This observation directly anticipates contemporary efforts to redesign pertussis vaccines using potent innate immune adjuvants that better mimic infection-induced signaling.

At the mucosal level, the interaction between *B. pertussis* and the respiratory epithelium remains a critical research focus. The bacterium exhibits a strong tropism for ciliated epithelial cells, and early colonization rapidly impairs ciliary function, as demonstrated in tracheal organ culture models [103]. Within hours of inoculation, *B. pertussis* expresses hemadsorptive fimbriae, closely associates with specialized epithelial surface structures, and releases cytotoxic factors—including TCT—that damage both ciliated and non-ciliated cells [104]. These processes collectively undermine mucociliary clearance, establishing a permissive environment for persistent infection and transmission.

Human observational studies provide compelling evidence for the protective role of mucosal antibodies. In infants with pertussis, higher concentrations of *B. pertussis*–specific IgA in nasal secretions are inversely correlated with disease severity, supporting a central role for mucosal IgA in modulating clinical outcomes [105]. In animal models, both IgA and IgG have been shown to confer protection; however, they act at different stages of infection. Secretory IgA primarily interferes with bacterial attachment and epithelial colonization, whereas IgG acts earlier by neutralizing bacteria and toxins before stable adherence to the respiratory tract occurs [106]. These distinctions have profound implications for vaccine design, as current acellular vaccines induce strong systemic IgG responses but limited mucosal IgA, thereby protecting against severe disease without reliably preventing colonization or transmission.

Additional evidence suggests that cellular immunity contributes to control of *B. pertussis* beyond antibody-mediated mechanisms. The proliferation of intracellular or closely associated bacteria can be inhibited by lymphoid cells derived from fetal or immunoglobulin-depleted neonatal lymph nodes, indicating that non-antibody cellular mechanisms may restrict bacterial persistence [107]. Among *B. pertussis* products, leukoagglutinin activity—historically attributed to PT—has been identified as the primary factor capable of interfering with the bactericidal activity of normal human serum, leading to flocculation of a subset of the bacterial population in vivo [29]. This selective interaction suggests population heterogeneity during infection and raises important questions regarding bacterial subpopulations that may preferentially evade immune clearance.

## 14. Future Research Priorities

Collectively, these findings highlight several critical directions for future research. First, there is a need to better characterize how antibody pressure modulates *B. pertussis* phenotype and virulence gene expression during infection and following vaccination. Second, the differential roles of mucosal IgA, systemic IgG, and cellular immunity in preventing disease versus blocking transmission require further elucidation in both human studies and refined animal models. Third, understanding species-specific immune responses may inform the development of more predictive experimental systems for vaccine evaluation. Finally, integrating these immunological insights into vaccine design—particularly strategies that enhance mucosal immunity and infection-like innate signaling—remains a central challenge for achieving durable, transmission-blocking protection against pertussis.

## Figures and Tables

**Table 1:** Comparison of licensed and next-generation pertussis vaccine platforms with respect to immune correlates and prevention of colonization and transmission.

Platform (Typical Route)	Antigen Composition	Mucosal IgA	Th1/Th17	Airway T_RM	Prevents Colonization/Transmission	Intended Target Population	Monovalent or Polyvalent	Key Evidence (Selected)
Licensed acellular pertussis (aP; IM)	PT, FHA, PRN ± FIM2/3 [5,6]	Low	Weak (Th2-leaning with alum)	Limited	Poor (protection mainly against disease, not carriage)	Infants (DTaP), children, adolescents, adults, pregnant women (Tdap boosters) [3,5,6,7]	Polyvalent (combined with diphtheria and tetanus toxoids) [5,6]	Strong serum anti-toxin IgG and reduced severe disease but limited mucosal immunity and weak Th1/Th17; does not reliably block carriage/transmission [33,70,71].
OMV-based pertussis vaccines (IN or IM; often IN in next-gen designs)	Multiple native outer membrane antigens (including PT, FHA, PRN, LPS components) [74,75]	High with intranasal delivery	Robust Th17 (and Th1) in preclinical models	Airway-local T-cell memory (preclinical)	Improved; intranasal OMV prevented nasal and lung colonization in mice	Primarily infants and children (future use), also potential boosters in adolescents/adults [74,75]	Monovalent (pertussis-focused)	Intranasal OMV vaccination induced mucosal IgA and Th17 responses and prevented colonization in both nasal cavity and lungs in mice [7].
BPZE1 live-attenuated nasal vaccine (IN)	Live attenuated *B. pertussis* expressing full antigen repertoire (genetically detoxified PT, ACT) [76,77]	High (mucosal + systemic responses)	Th1/Th17-skewed, infection-like programming	Expected strong airway T_RM (supported in models)	Yes/strong signal; reduces or prevents colonization in controlled settings	Currently adults in clinical trials; future use may include adolescents and children [76,77]	Monovalent (pertussis-only live vaccine)	Human trials show acceptable safety and immunogenicity [77]; a controlled human infection model phase 2b study showed that a single intranasal dose could prevent or substantially reduce colonization by virulent *B. pertussis* and markedly lower bacterial burden versus placebo.
Intranasal subunit strategies (e.g., aP antigens reformulated for IN delivery ± PRR agonists)	PT, FHA, PRN ± novel adjuvants (e.g., TLR agonists) [71,72,73,74]	Moderate-High (route-dependent)	Improved when paired with innate-programming adjuvants (e.g., TLR agonists)	Potential to seed airway memory (data emerging)	Partial; reduction in upper-airway burden in animals; clinical evidence limited	Potentially all age groups (infants, children, adults) depending on formulation [71,72,73,74]	Monovalent (pertussis-focused; may be combined in future formulations)	Intranasal delivery of acellular antigens can elicit mucosal IgA and IL-17A and reduce bacterial burden in upper and lower airways in mice, highlighting the importance of route and adjuvanting to restore mucosal correlates [2,73].

Overall, platforms that deliver antigens to the nasal mucosa and provide infection-like innate cues (OMV formulations and live-attenuated BPZE1) most consistently induce the combination of sIgA, Th17-associated mucosal immunity, and airway-local memory associated with control of colonization, whereas standard IM aP vaccination predominantly generates systemic antibodies that mitigate disease severity but leave residual carriage and transmission.

**Table 2:** Currently available pertussis vaccines and their principal characteristics.

Vaccine Category	Representative Formulations	Pertussis Antigen Composition	Typical Target Population	Monovalent or Polyvalent	Key Immunological Features
Whole-cell pertussis vaccine (wP)	DTwP-based combinations used in many national infant programs	Inactivated whole *B. pertussis* cells [1,2]	Infants and young children	Polyvalent	Broad antigenic exposure; stronger innate stimulation; generally more Th1/Th17-skewed and more durable immune priming than aP, but with greater reactogenicity
Acellular pediatric vaccine (DTaP), 3-component	Infanrix and related combinations	PT, FHA, PRN [5,6]	Infants and children <7 years	Polyvalent	Strong serum antibody responses with improved tolerability; less reactogenic than wP; limited mucosal immunity and faster waning
Acellular pediatric vaccine (DTaP), 5-component	Daptacel and related combinations	PT, FHA, PRN, FIM2, FIM3 [5,6]	Infants and children <7 years	Polyvalent	Broader antigen content than 3-component aP; strong humoral immunogenicity, but still does not reliably prevent colonization or transmission
Acellular booster vaccine (Tdap), 3-component	Boostrix	PT, FHA, PRN (reduced antigen content)	Adolescents, adults, pregnancy	Polyvalent	Booster formulation with reduced antigen load; enhances circulating antibodies and provides maternal passive protection to infants when given in pregnancy
Acellular booster vaccine (Tdap), 4-component	Adacel	PT, FHA, PRN, FIM2/3 (reduced PT/diphtheria content)	Adolescents and adults	Polyvalent	Booster humoral immunity with broader antigen content than 3-component Tdap; protection remains limited by waning and modest mucosal/cellular recall

**Table 3:** Risk stratification and therapeutic options in high-MRBP settings.

Population/Scenario	Preferred Agent(s) When MRBP Suspected/Confirmed	Key Safety Constraints	Role of Susceptibility Testing	Notes in High-MRBP Regions
Neonate (<1 month)	Azithromycin remains the only guideline-endorsed macrolide option; if MRBP confirmed, individualized management with specialist input	TMP–SMX contraindicated <2 months; erythromycin linked to infantile hypertrophic pyloric stenosis; limited safety/efficacy data for alternatives	Prioritize rapid genotyping (A2047G) + culture/qPCR where feasible	If MRBP is confirmed, there is no well-validated oral alternative; focus on supportive care, infection control, and expert consultation; consider investigational/locally recommended parenteral options only within protocolized care [66,97,98]
Infant (1–<2 months)	Azithromycin (standard of care); if MRBP confirmed, specialist-led individualized approach	TMP–SMX not recommended <2 months; tetracyclines/fluoroquinolones generally avoided	Genotype/culture to confirm MRBP whenever possible	Highest-risk group for severe disease; hospital monitoring often warranted; prioritize eradication of household source cases [66,97,98]
≥2 months (children/adolescents)	TMP–SMX (alternative agent when MRBP suspected/confirmed)	Avoid in sulfonamide allergy; monitor for adverse effects; avoid in late pregnancy if adolescent is pregnant	Genotyping useful to avoid ineffective macrolides; phenotypic MIC testing may support local surveillance	If TMP–SMX cannot be used, evidence for alternatives is limited; some reports suggest in vitro/in vivo activity of certain β-lactam/β-lactamase inhibitor combinations, but these are not standard guideline therapies [54,57,98,100]
Adults (non-pregnant)	TMP–SMX when MRBP suspected/confirmed; macrolides only if susceptibility likely/confirmed	Contraindications as above; consider drug interactions	Genotyping/culture where available to guide therapy and prophylaxis	For non-severe disease, the main antibiotic goal is to reduce transmission; if MRBP is circulating, public-health measures and targeted prophylaxis strategies become more important [54,57,98,100]
Pregnancy/immediate postpartum	Macrolide (azithromycin) is typically preferred when susceptible; in high-MRBP settings, prioritize susceptibility testing and individualized risk–benefit decisions	TMP–SMX generally avoided (especially 1st trimester and near term); tetracyclines/fluoroquinolones generally avoided in pregnancy	Rapid genotyping is particularly valuable to avoid prolonged ineffective therapy	Because robust MRBP-specific pregnancy-safe alternatives are lacking, prevention (maternal vaccination) + early case identification and infection control are critical; consult obstetrics/ID for any non-standard regimens [97,98,99]
Immunocompromised hosts	Treat early; TMP–SMX may be used when MRBP suspected/confirmed if not contraindicated	Higher risk of complications; drug–drug interactions common; monitor closely	Lower threshold for microbiologic confirmation and follow-up sampling	Consider longer observation and follow-up to ensure clearance; integrate with infection control to reduce nosocomial transmission [54,98,99,100,101]

## Data Availability

All data supporting the findings of this study are included within the manuscript.
